# The Finances of a Welsh Hospital

**Published:** 1910-02-05

**Authors:** Leonard D. Rea

**Affiliations:** (Secretary and General Supt.). The Infirmary, Cardiff


### Editor's Letter-Box.

### THE FINANCES OF A WELSH HOSPITAL.

*To the Editor of* The Hospital.

Sir,—I thank you for the kind hospitality afforded in your columns to my letter of the 22nd inst., and I am glad that in your commentary headed "Hospital Finance in Wales" you indicate the points not elucidated in my letter. I am not at liberty to make public the arrangements which we have with our bankers, but I may say that under present circumstances the infirmary stands to gain by having invested the £40,000. We have been able to obtain for it practically the same rate of interest as that charged by the bank. Moreover, investment sooner or later would have been necessary, as the money had been given specifically for that purpose, and, as a matter of fact, a considerable proportion had already been invested by the donors, the deeds of the securities being handed over to the infirmary trustees. Another feature, of a temporary character, arises from the circumstance that the amount of the overdraft is more than balanced by that placed to the infirmary account for the purpose of building the new wing and extensions. Satisfactory arrangements have been made with the bankers regarding the interest on this (which is, of course, credited to the New Wing Fund), but similar kindly treatment would not be extended to any credit balance exceeding the amount of the overdraft.

The reference to local experience applies to many years ago when an existing overdraft was defrayed by a special appeal, but no provision was then made to prevent the recurrence of the overdraft which was entirely due to the excess of expenditure over income. The result was that within a few years the overdraft had again assumed similar proportions, when it was found more difficult to obtain subscriptions for its reduction, as the public did not understand why the infirmary was suffering from a financial difficulty which, in their opinion, had been removed within recent years. We find that the public are prone to think that when they have removed a debt the hospital requires no further financial sacrifice on their part. The fact was, and is, that the overdraft has never been our difficulty; the real difficulty is what we are facing at present—the inadequacy of the yearly income.

The amount of overdraft quoted by Lord Aberdare relates to an estimate framed for the year 1910, based upon the income and expenditure for 1909, no margin being allowed for reduction in cost or increase of income. These figures were prepared only for the information of the treasurer and the bankers, and were necessarily pessimistic.

Pray accept my apologies for so far encroaching on your valuable space, and believe me, Sir,

Your obedient servant,

Leonard D. Rea (Secretary and General Supt.).

The Infirmary, Cardiff, January 31, 1910.

